# Pilot Study to Evaluate the Efficacy of Polynucleotide Sodium Compared to Sodium Hyaluronate and Crosslinked Sodium Hyaluronate in Patients with Knee Osteoarthritis

**DOI:** 10.3390/jcm10051138

**Published:** 2021-03-09

**Authors:** Ji Yeong Kim, Yoo Na Kim, Yu Jung Lee, Sung Eun Sim, Yu Ri Ko, Jin Woo Shim, Ku Sang Lee, Mina Joo, Hue Jung Park

**Affiliations:** 1 Department of Anesthesiology and Pain Medicine, Seoul St. Mary’s Hospital, College of Medicine, The Catholic University of Korea, Seoul 06591, Korea; apple_queen@naver.com (J.Y.K.); euny62827@catholic.ac.kr (S.E.S.); finocchio@hanmail.net (Y.R.K.); hisdoctorjwshim@gmail.com (J.W.S.); hikusang@gmail.com (K.S.L.); jma1128@naver.com (M.J.); 2 Mapo Hapjung Bone Orthopedics, Seoul 06591, Korea; yoonayaaa@gmail.com; 3 Seoul Bone Pain Clinic, Seoul 06591, Korea; dasaki7@gmail.com

**Keywords:** crosslinked sodium hyaluronate, intra-articular injection, knee osteoarthritis, polynucleotide sodium, sodium hyaluronate

## Abstract

Degenerative arthritis of the knee joint has become a major social problem worldwide due to population aging. There are several treatment options for knee osteoarthritis, and the intraarticular injection of sodium hyaluronate is commonly selected by many clinicians as a nonsurgical treatment. However, the efficacy of the treatment is controversial. In this pilot study, we aimed to compare polynucleotide sodium (Conjuran^®^) with sodium hyaluronate (Hyruan Plus^®^) and 1,4-butanediol diglycidyl ether-crosslinked sodium hyaluronate (Synovian^®^) in terms of analgesic efficacy after intraarticular injection in patients with knee osteoarthritis. One of the three intraarticular agents was selected according to what agents were available for outpatients when each patient was enrolled in the study. The 15 enrolled patients were subdivided into 3 groups of 5 patients each. Three injections were performed under ultrasound guidance at a 1-week intervals over a total of 3 weeks. The visual analog scale (VAS) score, the Korean version of the Western Ontario and McMaster Universities Arthritis Index (K-WOMAC), the EuroQol five-dimension scale (EQ-5D) score, and the Korean version of the painDETECT Questionnaire (K-PDQ) score were evaluated before injection and at 1, 2, and 6 weeks after the start of the treatment protocol. The primary endpoint was the change in weight-bearing pain at 4 weeks after the last injection. Secondary endpoints included pain at rest and during walking and the K-WOMAC, EQ-5D, and K-PDQ scores. Weight-bearing pain decreased significantly more from pretreatment to 6 weeks after the start of the treatment protocol in the polynucleotide sodium-treated patients than in the patients who were treated with other agents (*p* = 0.006, one-way ANOVA). There were no significant between-group differences in the other secondary endpoints. No adverse events occurred. In conclusion, polynucleotide sodium could effectively reduce weight-bearing pain in the patients with knee osteoarthritis compared to standard hyaluronic acid viscosupplementation.

## 1. Introduction

Osteoarthritis (OA) is a degenerative disease of the synovial joints that has a high prevalence and causes severe pain and disability, resulting in decreased quality of life [1]. The main features are erosion of articular cartilage and changes in synovial fluid characteristics, resulting in decreased elastoviscosity [2]. Nonpharmacological treatments for OA in overweight patients include weight loss and exercise [3]. Pharmaceutical treatments include oral acetaminophen, nonsteroidal anti-inflammatory drugs (NSAIDs), intraarticular steroid injections, and sodium hyaluronate injections [4]. Various factors, such as arthritis severity, patient medical conditions, and patient preferences, should be considered comprehensively in the selection of a treatment method. If OA is seriously symptomatic, there is no response to medical therapy, and activities of daily living are persistently restricted, the patient should be transferred to orthopedic surgery. Medical conditions that should be considered in the selection of a treatment for OA include high blood pressure, cardiovascular disease, gastrointestinal bleeding risk, and chronic renal failure. Service availability, such as insurance coverage, can also influence the choice of physical and psychological services [3,5].

Sodium hyaluronate is a pharmaceutical treatment that has been commonly used for moderate OA in recent years [6,7]. Because the concentration and chain length of synovial hyaluronan have been observed to be low in OA patients, intraarticular injections of sodium hyaluronate improve shock absorption by replenishing sodium hyaluronate in the joint cavity [8,9,10]. Unfortunately, the effects of these injections are not significantly greater than those of placebos, according to recent reports [3,11,12,13]. When prior systematic reviews were reanalyzed in consideration of the risk of bias in individual studies, a benefit was evident only among studies with a higher risk of bias. In trials with a low risk of bias, the effect size of hyaluronic acid injections compared to saline injections was zero [13]. Zhang et al. stated that the effect of hyaluronic acid injections constituted a contextual effect [14]. In clinical practice, the decision to use hyaluronic acid injections may be regarded more favorably than offering no intervention for knee OA patients whose response to nonpharmaceutical treatment has been insufficient [14].

An intraarticular injection agent that can be used as an alternative to sodium hyaluronate is crosslinked sodium hyaluronate, which has a higher molecular weight and greater stability and viscosity than unmodified sodium hyaluronate due to crosslinking. Therefore, this crosslinked agent may be expected to have a longer duration of action than hyaluronate [15,16,17]. Another alternative that has recently emerged is polynucleotide sodium, which has a controlled natural origin (fish sperm) and is a highly purified agent [18]. Studies comparing sodium hyaluronate and polynucleotide sodium [5,18,19] or comparing sodium hyaluronate and crosslinked sodium hyaluronate have been conducted [7]. However, there have been no reported studies comparing all three drugs at the same time. The purpose of this pilot study was to evaluate the efficacy of the three drugs simultaneously by analyzing data from groups of patients with knee OA who each received one of the three drugs intraarticularly at a pain center.

## 2. Methods and Materials

### 2.1. Study Design

In this pilot study, we retrospectively reviewed the medical records of 15 patients who met the inclusion and exclusion criteria and whose knee joints had been treated with intraarticular knee joint injections at the pain center of Seoul St. Mary’s Hospital from June 2020 to August 2020. The 15 enrolled patients were subdivided into 3 groups of 5 patients each, and they received 3 intraarticular injections in the affected knee of polynucleotide sodium, (group C, Conjuran^®^ group, *n* = 5) sodium hyaluronate (group H, Hyruan Plus^®^, *n* = 5) or cross-linked sodium hyaluronate (group S, Synovian^®^, *n* = 5) at a 1-week intervals. One of the three intraarticular agents was selected according to what agents were available for outpatients when each patient was enrolled in the study. This clinical trial was approved by the Institutional Review Board of Seoul St. Mary’s Hospital (KC20RISI0659), and was registered at http://www.clinicaltrials.gov/ on 29 October 2020 (2020-2733-0001). The requirement for patient consent was waived due to the retrospective nature of the study, which used only formal electronic medical records. This study was conducted in accordance with the Declaration of Helsinki.

### 2.2. Materials

The polynucleotide sodium used in this study (Conjuran^®^, Pharma Research Products, Gangneung, Korea) was colorless and viscoelastic and was provided in prefilled sterile syringes containing a solution of 2 mL of (concentration 20 mg/mL). Crosslinked sodium hyaluronate with a molecular weight >10,000 kDa (Synovian^®^, LG Life Sciences, Seoul, Korea) was provided in a prefilled sterile syringe containing 60 mg of crosslinked hyaluronic acid in 3 mL of a solution. Linear high molecular weight hyaluronate sodium with a mean molecular weight of 3000 kDa (Hyruan Plus^®^, LG Life Sciences, Seoul, Korea) was provided in a prefilled sterile syringe containing 20 mg of hyaluronic acid in 2 mL of a solution.

### 2.3. Study Population

Patients aged 40 years or older with Kellgren-Lawrence grades [20] I–III knee OA as determined by X-ray examination were enrolled. All patients underwent simple radiography, including standing anteroposterior and lateral views, a standing posteroanterior view under 45° of knee flexion and the merchant view. The inclusion criteria were as follows: Kellgren-Lawrence grade I–III OA as determined by X-ray examination; weight-bearing pain ≥40 mm in the affected knee on a 100-mm VAS; and persistent pain despite pharmacological therapy and/or physiotherapy. The exclusion criteria were as follows: (1) a history of severe trauma or fracture; (2) rheumatoid arthritis or another type of metabolic arthritis; (3) infection (osteomyelitis) around the joint; (4) a history of knee joint replacement surgery or intervention; (5) hip OA; (6) severe pain, such as complex regional pain syndrome, Paget’s disease, disc herniation, gout, and recurrent pseudogout; (7) complete loss of the femoral knee gap on X-ray examination; and (8) use of anticoagulants or platelet aggregation inhibitors within one week prior to the baseline assessment.

### 2.4. Experimental Intervention

**Figure 1:**
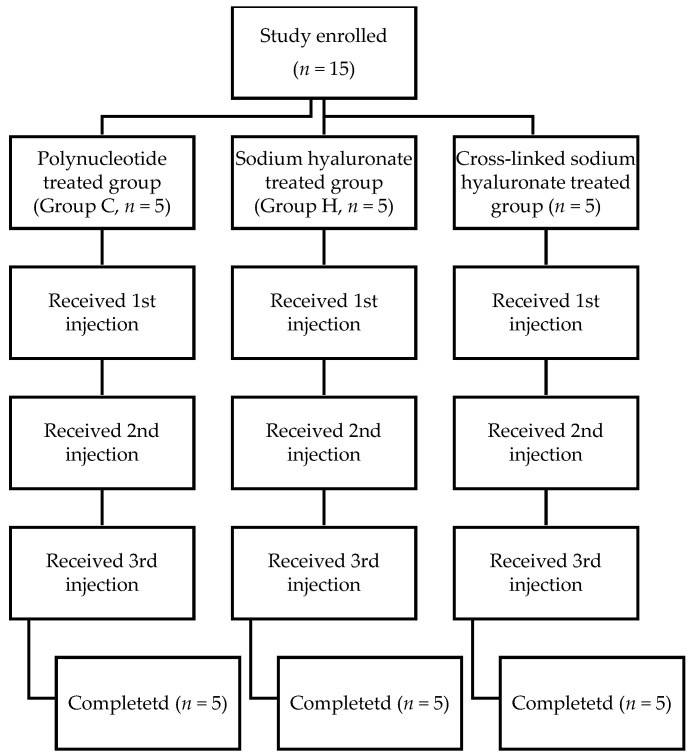
Diagram of the study protocol.

All patients were given injections three times at an intervals of 1 week. The first injection was performed at the beginning of the treatment (T0 = before injection), the second injection was performed one week later (T1), and the third injection was performed two weeks later (T2). Then, the patients returned for a clinical follow-up four weeks after the end of the protocol (T6). All clinical parameters were evaluated immediately before the first treatment (T0), after each subsequent injection (T1, T2), and at the final follow-up (T6). The protocol of the study is shown in Figure 1. All injections were performed under ultrasound guidance. The prescribing doctor, the doctor filling out the questionnaire, and the doctor performing the procedure were all different individuals. In group C, one 2-cc volume of polynucleotide sodium was injected once a week, and a total of three intraarticular injections were performed. In group H, one 2-cc volume of sodium hyaluronate was injected once a week, and a total of 3 intraarticular injections were performed. In group S, one 3-cc volume of crosslinked sodium hyaluronate was injected once at the first visit in the knee joint. Then, at the second and third visits, in group S, the same volume of normal saline was injected into the intraarticular space. A neurological examination was performed after the procedure in all patients. Before discharge, each patient was repeatedly examined to confirm that no abnormalities had occurred; the patient was released only if no abnormalities were observed.

### 2.5. Outcome Measurements

The primary outcome of the study was the change in the VAS (100 mm) score for knee pain under weight bearing from T0 to T6. The secondary outcomes included the changes in the VAS score [21] for knee pain at rest and during walking and in the K-WOMAC score [22], EQ-5D score [23] and K-PDQ score [24] from T0 to T6. Prior to the injection at each outpatient visit, the physician in charge of the questionnaire asked the patient each question of the questionnaire and recorded the answer. Safety outcomes were estimated in terms of adverse events. All of the adverse events occurring during the entire follow-up period were recorded at every visit according to the physician’s direct observation and as determined by asking the patients if any adverse events had occurred. Vital signs were recorded and laboratory tests were performed, and any clinically significant changes during the study were recorded.

### 2.6. Statistical Analysis

For statistical analysis, SPSS Statistics (IBM^®^, Armonk, NY, US) was used, and the two-way significance level was 0.05. A total of 15 patients were assessed, including 5 in each group. To compare the changes in each parameter from T0 to T6 among groups, one-way ANOVA was performed, and post hoc analysis was performed with Tukey’s honestly significant difference (HSD) test only when significant results were revealed by one-way ANOVA. Paired t tests were performed for intragroup comparisons.

## 3. Results

**Figure 2:**
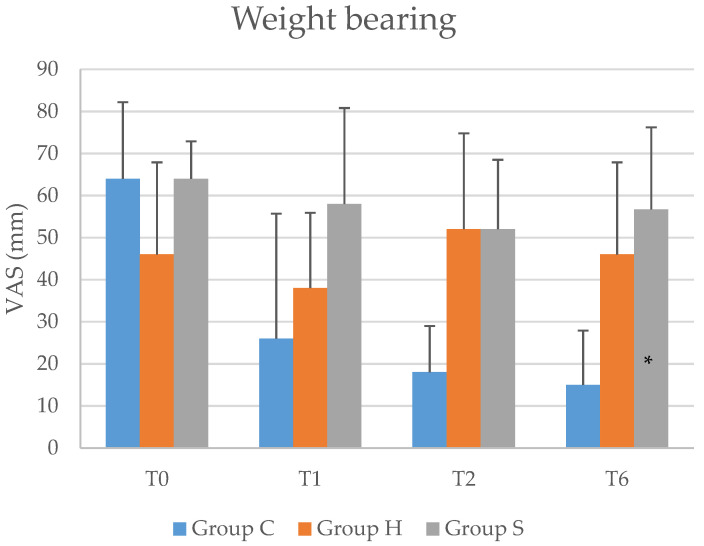
Mean differences from baseline in the VAS score under weight-bearing with the 95% confidence interval (*t*-test). VAS = visual analog scale; T0 = before injection; T1 = 1 week after the start of the protocol; T2 = 2 weeks after the start of the protocol; T6 = 6 weeks after the start of the protocol. Group C indicates the group of patients treated with polynucleotide sodium. Group H indicates the group of patients treated with sodium hyaluronate. Group S indicates the group of patients treated with crosslinked sodium hyaluronate. The *p* values are based on the results of one-way ANOVA comparing Diff (T6–T0) among the groups; * *p* < 0.05.

Table 1 shows the basic demographic data and there were no differences among groups. Table 2 shows the mean ± standard deviation of the VAS scores in each group under weight bearing at T0 and T6 and the *p* values for the between-group comparisons of the change in the score from T0 to T6. Table 3 shows the results of post hoc analysis using Tukey’s HSD test. Figure 2 shows the mean differences with respect to baseline as well as the 95% confidence intervals for the VAS score under weight-bearing in groups C, H and S. One patient in group C and two patients in group S did not visit the clinic within the last six weeks. Missing values were not separately processed.

### 3.1. Primary Results

The change in the weight-bearing pain score from T0 to T6 significantly differed among the three groups (one-way ANOVA, *p* = 0.026). On post hoc analysis, the VAS score for weight-bearing pain decreased more from T0 to T6 in group C than in group H (Tukey’s HSD test, *p* = 0.021).

### 3.2. Secondary Results

The change in the pain score at rest from T0 to T6 also significantly differed among groups (one-way ANOVA, *p* = 0.011). According to the post hoc analysis, the VAS pain score at rest decreased more from T0 to T6 in group C than in group H (Tukey’s HSD test, *p* = 0.014). The changes in the K-WOMAC and EQ-5D scores over time did not differ among the three groups.

In addition, when an intragroup comparison was performed with a paired t test and Bonferroni’s correction, only the weight-bearing VAS pain score, the total K-WOMAC score, and EQ-5D mobility score significantly decreased from T0 to T6, and only in group C. When a total K-PDQ score of over 19 was classified as neuropathic pain [24], there were no patients in group C, one patient in group H, and one patient in group S with neuropathic pain. In both of these patients, the K-PDQ score decreased to 13 points or less at the follow-up conducted one month after the completion of the treatment protocol. No adverse events occurred during the entire follow-up period.

## 4. Discussion

This study shows that polynucleotide sodium could be beneficial, as it resulted in a comparable improvement in the weight-bearing pain in patients with knee OA as sodium hyaluronate and crosslinked sodium hyaluronate.

The strength of our clinical study is that it is to compares polynucleotide sodium and crosslinked sodium hyaluronate. Previous randomized controlled trials have been conducted to compare polynucleotide and sodium hyaluronate in the form of intraarticular injections in terms of their efficacy and safety in treating knee OA [5,18,19]. Regarding the analgesic effect of polynucleotide compared with that of sodium hyaluronate, the severity of ‘weight-bearing pain’ and ‘resting pain’ improved significantly more at 6 weeks after the start of treatment in the group treated with polynucleotide sodium than in the group treated with sodium hyaluronate. However, there were no differences between the group treated with polynucleotide sodium and the group treated with crosslinked sodium hyaluronate regarding the changes in different clinical indicators from before treatment to 6 weeks after the start of treatment.

The strength of polynucleotides suggested in previous papers was that they could not only provide mechanical supplementation (sodium hyaluronate) but also cause physiologic changes [5,25]. The mechanism of action polynucleotides is believed to be that polynucleotides bind to large amounts of water and undergo structural rearrangemen by orienting water molecules to form a 3D gel [26,27,28,29]. Polynucleotides are degraded by enzymes and gradually release water molecules and oligonucleotides in the joint cavity [30]. Because polynucleotides are extracted from natural sources including fish sperm, enzymatic degradation products of polynucleotides are physiologically present in the extracellular environment and are useful materials for cells [31]. The resulting nucleotide, purine, and pyrimidine bases are used by cells to enhance metabolism and support the physiological process of cartilage repair [18,32,33]. We expected that polynucleotides would produce better clinical results than crosslinked sodium hyaluronate, but there was no difference in the clinical results.

We included evaluations of clinical aspects, including quality of life and neuropathic pain components. Contrary to our study, previous studies have evaluated only pain and function in patients with knee OA [5,18,19,32]. Concerning the between-group comparisons of the K-WOMAC and EQ-5D scores, there were no significant differences. Regarding the K-PDQ score, when patients with scores of at least 19 points were classified as having neuropathic pain and those with scores at most 13 points were classified as having nonneuropathic pain group [24], neuropathic pain was not present in any of the patients in the polynucleotide group, but 1 patient in the sodium hyaluronate group and 1 patient in the crosslinked sodium hyaluronate group exhibited neuropathic pain. The rest of the patients did not have neuropathic pain. Golob et al. reported that in knee OA patients, the pain is more severe when the PDQ score is within the neuropathic pain range [34]. The authors also stated that if there is neuropathic pain in knee OA patients, the treatment method should be different. In this pilot study, there were too few patients in the neuropathic pain group, so additional research is needed to determine whether intraarticular drug injections can reduce neuropathic pain components.

The limitations of this study are as follows: (1) the follow-up period was short; (2) the number of patients per group was small; (3) blinding was absent, and (4) no restrictions were imposed on the use of analgesic medications. In the future, a double-blinded clinical trial should be conducted to overcome these limitations. In addition, crosslinked sodium hyaluronate was injected only once, and the other two drugs were injected three times for comparison with each other, so there is concern about whether the pure effects of the drugs can be compared when the same number of injections of the medication were not administered. According to Korean insurance standards, crosslinked sodium hyaluronate can be applied up to once within six months, sodium hyaluronate can be applied up to 3 times within 6 months, and polynucleotides can be applied up to 5 times within 6 months. In clinical practice, crosslinked sodium hyaluronate can be injected only once, but if polynucleotide sodium is injected once and the effect is not sufficient, another injection may be given at the next visit. The protocol of the study reflects this clinical situation, and a similar design will be used in the planned double-blinded randomized controlled study in the future.

## 5. Conclusions

In this preliminary study, we showed that polynucleotide sodium could reduce weight-bearing pain more effectively 6 weeks after the start of treatment than sodium hyaluronate and crosslinked sodium hyaluronate in knee OA patients. To address the limitations imposed by the preliminary nature of this study, our center is conducting a randomized study with a larger sample size.

## Figures and Tables

**Table 1:** Basic demographic data.

	Group C (*n* = 5)	Group H (*n* = 5)	Group S (*n* = 5)	*p*-Value
Age (years)	68.0 ± 6.4	68.4 ± 8.2	67.6 ± 6.9	0.985
Sex				0.397
Male	1 (20%)	0 (0%)	0 (0%)	
Female	4 (80%)	5 (100%)	5 (100%)	
Weight (kg)	60.5 ± 4.8	64.3 ± 9.6	60.4 ± 11.2	0.747
Height (cm)	157.9 ± 4.7	154.7 ± 5.9	153.1 ± 7.7	0.481

Group C indicates the group of patients treated with polynucleotide sodium. Group H indicates the group of patients treated with sodium hyaluronate. Group S indicates the group of patients treated with crosslinked sodium hyaluronate. Continuous variables are shown as the mean ± standard deviation and categorical variables are shown as the number (percentage).

**Table 2:** Weight-bearing pain in all groups.

		VAS Score (mm)	95% Confidence Interval	*p*-Value
Group C (*n* = 5)	T0	64.0 ± 18.2		
	T6	12.0 ± 13.0		
	T6–T0	−52.0 ± 16.4	−72.4–−31.60	0.006 *
Group H (*n* = 5)	T0	44.0 ± 18.2		
	T6	46.0 ± 21.9		
	T6–T0	2.0 ± 22.8	−26.31–30.60	
Group S (*n* = 5)	T0	64.0 ± 8.9		
	T6	54.0 ± 19.5		
	T6–T0	−10.0 ± 26.5	−42.85–22.85	

VAS = visual analog scale; T0 = before injection; T6 = 6 weeks after the start of the protocol. Group C indicates the group of patients treated with polynucleotide sodium. Group H indicates the group of patients treated with sodium hyaluronate. Group S indicates the group of patients treated with crosslinked sodium hyaluronate. Data are shown as the mean ± standard deviation. The *p* values are based on the results of one-way ANOVA comparing Diff (T6–T0) among the groups; * *p* < 0.05.

**Table 3:** Between-group comparisons of the Diff (T6–T0) in weight-bearing pain.

Post Hoc Analysis (Tukey’s HSD Test)		95% Confidence Interval	*p*-Value
Group C	Group H	−54.0 (−91.6–−16.4)	0.006 *
	Group S	−42.0 (−79.6–−4.4)	0.029 *
Group H	Group S	12.0 (−25.6–49.6)	0.680

VAS = visual analog scale; HSD = honestly significant difference. Group C indicates the group of patients treated with polynucleotide sodium. Group H indicates the group of patients treated with sodium hyaluronate. Group S indicates the group of patients treated with crosslinked sodium hyaluronate. The *p* values are based on the results of post hoc analysis using Tukey’s HSD test comparing Diff (T6–T0) between the groups; * *p* < 0.05.

## Data Availability

The data presented in this study are available on request from the corresponding author.
